# Effect of Mild Hypoinsulinemia on Renal Hypertrophy: Growth Hormone/Insulin-Like Growth Factor I System in Mild Streptozotocin Diabetes

**DOI:** 10.1080/15604280214937

**Published:** 2002

**Authors:** Mogher Khamaisi, Allan Flyvbjerg, Ziv Haramati, Gadi Raz, Isaiah D. Wexler, Itamar Raz

**Affiliations:** 1 Diabetes Center and Department of Internal Medicine Hadassah University Hospital Jerusalem Israel; 2 Medical Research Laboratories Aarhus University Hospital Aarhus Denmark; 3 Diabetes Center and Department of Internal Medicine Hadassah University Hospital-EinKerem P.O.B. 12000 Kiryat Hadassah Jerusalem 91120 Israel

## Abstract

The metabolic aberrations associated with diabetes mellitus profoundly alter the growth hormone/insulin-like growth factor I (GH/IGF-I) system. In severe experimental diabetes, serum IGF-I level is reduced, reflecting altered hepatic expression. On the other hand, increased levels of kidney IGF-I have been implicated in the development of diabetic kidney disease. This study aimed to examine the effect of mild experimental diabetes with hypoinsulinemia on both the systemic and renal GH/IGF-I systems in a low-dose streptozotocin (STZ)-induced diabetic rat. Diabetic animals with mild hypoinsulinemia developed renal hyperfiltration within 3 days of diabetes, whereas the renal size increased significantly only between 30 and 48 days of diabetes. Plasma GHlevels were unchanged during the entire course of the study, but a decrease in serum IGF-I, IGF-binding protein 3 (IGFBP-3), and IGF-binding protein 4 (IGFBP-4) occurred after 10, 30, and 48 days. Kidney IGF-I and IGF-binding protein 1 (IGFBP-1) mRNA expression increased after 10 and 30 days of diabetes. A significant increase in kidney IGFBP-1/2, IGFBP-3, and IGFBP-4 proteins was seen after 48 days of diabetes.Apositive correlations was found between renal growth and insulin/glucose ratio (*r* = .57), kidney IGF-I (*r* = .57), IGFBP-1 mRNA(*r* = .43), IGFBP-1/2 (*r* = .41), and IGFBP-4 levels (*r* = .40). These results demonstrate hyperfiltration within 3 days of diabetes and a similar response in the IGF-I system in mildly and severely hypoinsulinemic rats; however, renomegaly develops slower in mildly diabetic rats at least partly due to delayed changes in the renal IGF and IGF BPs.


*Int. Jnl. Experimental Diab. Res.*, 3:257–264, 2002
Copyright © 2002 Taylor & Francis
1560-4284/02 $12.00 + .00
DOI: 10.1080/15604280290014008

Taylor & Francis healthsciences

### Effect of Mild Hypoinsulinemia on Renal Hypertrophy: Growth Hormone/Insulin-Like Growth Factor I System in Mild Streptozotocin Diabetes

**Mogher Khamaisi,[1] Allan Flyvbjerg,[2] Ziv Haramati,[1] Gadi Raz,[1] Isaiah D. Wexler,[1] and Itamar Raz[1]**

[1]*Diabetes Center and Department of Internal Medicine, Hadassah University Hospital, Jerusalem, Israel*
[2]*Medical Research Laboratories, Aarhus University Hospital, Aarhus, Denmark*

**The metabolic aberrations associated with diabetes mellitus profoundly alter the growth hormone/insulin-like growth factor I (GH/IGF-I) system. In severe experimental diabetes, serum IGF-I level is reduced, reflecting altered hepatic expression. On the other hand, increased levels of kidney IGF-I have been implicated in the development of diabetic kidney disease. This study aimed to examine the effect of mild experimental diabetes with hypoinsulinemia on both the systemic and renal GH/IGF-I systems in a low-dose streptozotocin (STZ)-induced diabetic rat. Diabetic animals with mild hypoinsulinemia developed renal hyperfiltration within 3 days of diabetes, whereas the renal size increased significantly only between 30 and 48 days of diabetes. Plasma GH levels were unchanged during the entire course of the study, but a decrease in serum IGF-I, IGF-binding protein 3 (IGFBP-3), and IGF-binding protein 4 (IGFBP-4) occurred after 10, 30, and 48 days. Kidney IGF-I and IGF-binding protein 1 (IGFBP-1) mRNA expression increased after 10 and 30 days of diabetes. A significant increase in kidney IGFBP-1/2, IGFBP-3, and IGFBP-4 proteins was seen after 48 days of diabetes. A positive correlations was found between renal growth and insulin/glucose ratio ($r = .57$), kidney IGF-I ($r = .57$), IGFBP-1 mRNA ($r = .43$), IGFBP-1/2 ($r = .41$), and IGFBP-4 levels ($r = .40$). These results demonstrate hyperfiltration within 3 days of diabetes and a similar response in the IGF-I system in mildly and severely hypoinsulinemic rats; however, renomegaly develops slower in mildly diabetic rats at least partly due to delayed changes in the renal IGF and IGF BPs.**

**Keywords** Diabetic Kidney Disease; Growth Hormone; Hyperfiltration; Insulin-Like Growth Factor I; Renomegaly

Diabetes mellitus is frequently associated with cardiovascular and kidney complications. The pathogenic mechanisms of diabetic complications have not been fully elucidated, but levels of both hyperglycemia and insulinemia trigger a cascade of events leading to hemodynamic and morphology changes [1]. An active area of research has been to identify the mediators of kidney complications, and to determine the relationship between these mediators and the metabolic environment of diabetes.

The search for specific mediators for the diabetic nephropathy has been focused on the growth hormone/insulin-like growth factor (GH/IGF) system [2–4]. In the diabetic state, alterations of GH and the components of the IGF-I system have been found in most tissues, including liver, kidney, retina, and endothelium. The response of the IGF-I system to diabetes varies among tissues. Circulating levels of IGF-I, representing primarily liver production, decline in the diabetic state, whereas there is a significant increase in IGF-I in the kidney almost immediately after the onset of diabetes [5–7]. IGF-I is a renotrophic factor implicated in the development of diabetic kidney disease, and it has been shown that IGF-I accumulation is necessary for renomegaly as an early feature of diabetic nephropathy [8].

A family of IGF-binding proteins (IGFBPs) modulates the effects of IGF-I. Levels of the different IGFBPs also change in

Received 15 August 2002; accepted 10 October 2002.
This study was supported in part by the Ministry of Sciences, Israel, and the Danish Medical Research Council (grant no. 9700592). Dr. Khamaisi is partly supported by a grant from the Israeli Diabetes Association.
Address correspondence to Mogher Khamaisi, M.D. Ph.D., Diabetes Center and Department of Internal Medicine, Hadassah University Hospital-Ein Kerem, P.O.B. 12000, Kiryat Hadassah, Jerusalem, Israel 91120. E-mail: murir@hadassah.org.il diabetes, with IGFBP-1 increasing in the plasma and renal cortex while others declining (IGFBP-3 in plasma and IGFBP-4 in plasma and kidney) [2]. The precise role of the IGFBPs as mediators of diabetic kidney disease and other diabetic complications has not yet been clarified. It has been suggested that some of these factors may potentiate the effect of IGF-I by ligation of IGF-I to cell surface receptors [2].

The alterations in the GH-IGF axis have been related to the severity of diabetes. In short-term streptozotocin (STZ)-induced diabetes, changes in the plasma and renal levels of IGF are related to the dose of STZ administered to the rats [9]. Both high blood glucose and low insulin levels have been related to the changes in the IGF system in severe diabetes [6, 10–12]. However, the role of each of these factors have not been clearly defined, as it has been difficult to distinguish between specific effects of insulin and glucose. In the present study, we analyzed the effects of mild hypoinsulinemia on the GH-IGF axis over a period of 7 weeks and its impact on the development of diabetic kidney disease. In this model of hypoinsulinemia, hyperglycemia is limited to the nonfasting state; both insulin and glucose levels are close to normal after overnight fasting. Our results indicate that the same pattern of changes in the GH-IGF axis seen in severe diabetes occurs also in mild diabetes, but at a later stage. In addition, there was a temporal discrepancy between hyperfiltration and development of renal hypertrophy in mild hypoinsulinemic STZ rat, with the latter being dependent on changes in the IGF system.

### MATERIALS AND METHODS

### Animals

Male Hebrew University Sabra rats, weighing 190 to 210 g, were used. The animals were maintained and treated in accordance with the regulations of the Hadassah-Hebrew University Center Committee on Animal Care and Use. Rats were housed 3 per cage in a room with 12:12-hour artificial light cycle, temperature 21°C ± 2°C and humidity 55% ± 2%. Mild hypoinsulinemic, hyperglycemic diabetic state was induced by a single intraperitoneal injection of freshly prepared STZ solution (50 mg/kg body weight diluted in dextrose 5%, pH 4.5). To be included in the analysis, animals that received STZ had to have a fasting blood glucose level $<$6.7 mM on 2 occasions, monitored with portable glucose-measuring device (Glucometer Elite, Bayer, Elkhart, IN, USA) and a nonfasting blood glucose level $>$13.9 mM.

Throughout the study, all animals were maintained on standard laboratory rat chow and provided water ad libitum. At 3, 10, 30, and 48 days after the induction of diabetes, 8 diabetic and 8 age-matched control rats were placed in metabolic cages, during which 24-hour urine samples were collected. Blood sample was also taken from the tail vein. At the end of the study, the rats were anaesthetized with an intraperitoneal injection of sodium phenobarbital, and blood was drawn from the aorta into heparin-containing tubes. Kidneys were rapidly removed, weighed, immediately frozen in liquid nitrogen, and kept at −70°C until analyzed.

### Analytic Procedures

Plasma glucose concentration was measured by the glucose oxidase method. Plasma insulin levels were determined by radioimmunoassay (RIA) using human insulin as a standard (Sorin Biomedica, Saluggia, Italy). Plasma and urine creatinine levels were determined by a standard automated application of the Jaffe reaction [13]. Creatinine clearance was calculated by standard laboratory method. Plasma GH was measured by RIA (Amersham International, UK). Serum IGF-I was measured after extraction in ethanol–acetic acid as previously described [6, 9]. Intra- and interassay coefficients of variations were $<$5% and $<$10%, respectively, for all assays.

### Kidney IGF-I Extraction and Radioimmunoassay

IGF-I extraction was performed according to the method of D'Ercole as previously described [6, 9, 10]. Briefly, kidneys were homogenized in 1 mM acetic acid with an Ultra Turrax TD25 and further disrupted using a Potter Elvehjelm homogenizer. The tissues were extracted twice. The sample was lyophilized and subsequently redissolved in a 40 mM phosphate buffer, pH 8.0. Tissue extracts were kept at −20°C until the IGF-I assay was performed. A linear relationship was found between biosynthetic IGF-I and extracted IGF-I immunoreactivity at different concentrations. The measured kidney IGF-I concentrations were all corrected for IGF-I in serum entrapped in the kidney tissue as previously described [11, 14].

### Western Ligand Blotting (WLB) for IGFBPs in Kidney and Serum

Approximately 50 mg of thawed kidney was homogenized as previously described [15, 16]. Protein content was determined by a commercial protein assay (Pierce Rockford, Rockford, IL) using bovine serum albumin as a standard. Aliquots containing 200 $\mu$g of protein, or in a subset of experiments 2 $\mu$L of plasma, were electrophoresed in a 10% sodium dodecyl sulfate–polyacrylamide gel electrophoresis (SDS-PAGE) under nonreducing conditions, and separated proteins were transferred by electroelution to nitrocellulose paper (Schleicher and Schuell, Munich, Germany). Membranes were incubated with approximately $0.5 \times 10^6$ cpm [$^{125}$I]-IGF-II (specific activity 2000 Ci/mmol, Amersham, UK) as detailed previously. Membranes were washed Tris-HCl buffer (TBS) containing 0.1% Tween, and the nitrocellulose were autoradiographed with Kodak x-ray. Specificity of the IGFBP band was confirmed by competitive coincubation with unlabeled IGF-II (Bachem, Bubendorf, Switzerland). WLBs were quantified by densitometry using a Shimazdu CS-9001 PC dual-wavelength flying spot scanner.

### RNase Protection Analysis

RNase protection assays were performed essentially as described previously using probes to IGFBP-1 [16]. In brief, total RNA was prepared from kidney tissue utilizing a ready RNA kit according to the manufacturer's instructions (Bet Haemek, Biological Industries, Israel). RNA was quantified by ultraviolet (UV) absorbance spectrophotometry. RNase protection by hybridization was performed using 20 $\mu$g of total RNA according to established protocols [17] using propes to IGFBP-1 [18]. After hybridization, the samples were digested with RNase A and T1 (Ambion, Austin, TX), and the protected fragments extracted with phenol-chloroform and ethanol precipitated. The fragments were separated electrophoretically on an 8% polyacrylamide–8 mM urea denaturing gel. Autoradiograms from each gel were scanned by a densitometer (Molecular Dynamic, Sunnyvale, CA). Changes in signals are expressed as the ratio between experimental and control values.

### Statistical Analysis

Data are expressed as mean ±SE. Each diabetic group was compared with the age-matched control, and statistical significance between 2 groups was evaluated using unpaired 2-tailed Student's *t* test. Significance was set as $P$ value $<.05$.

### RESULTS

The metabolic parameters of animals injected with 50 mg STZ, which met the inclusion criteria, are listed in Table 1 together with the age-matched nondiabetic control animals. All experimental animals had elevated serum glucose levels in nonfasted state and near normal levels of glucose when overnight fasted. Insulin levels in nonfasting state were not significantly lower than the values obtained in control animals. However, the insulin/glucose ratio was significantly lower in diabetic animals, indicating that an inadequate amount of insulin was secreted in response to food intake. Compared with animals from earlier experiments performed in our laboratory that received a higher dose of 70 mg/kg STZ and had elevated fasting glucose and low insulin levels, the low dose of STZ did result in marked weight loss in diabetic rats only after 30 days (Table 1).

**TABLE 1**
Physical and biochemical parameters of the control and diabetic animals

| | 3 days | 10 days | 30 days | 48 days |
|---|---|---|---|---|
| Nonfasting glucose (mM) | | | | |
| Control | 9.2 ± 1.4 | 9.5 ± 1.6 | 8.7 ± 1.1 | 8.9 ± 1.0 |
| Diabetic | 21.8 ± 4.2* | 22.9 ± 6.2* | 22.9 ± 5.2* | 27.6 ± 5.9* |
| Fasting glucose (mM) | | | | |
| Control | ND | 4.9 ± 0.4 | 4.4 ± 0.3 | 6.5 ± 0.8 |
| Diabetic | ND | 5.9 ± 1.5 | 6.6 ± 0.6 | 5.1 ± 1.1 |
| Nonfasting insulin (mU/L) | | | | |
| Control | 45 ± 18 | 39 ± 15 | 49 ± 10 | 37 ± 13 |
| Diabetic | 29 ± 15 | 34 ± 23 | 29 ± 8 | 22 ± 7 |
| Insulin/glucose ratio | | | | |
| Control | 5.2 ± 2.9 | 4.0 ± 1.2 | 5.7 ± 1.3 | 4.8 ± 2.3 |
| Diabetic | 1.5 ± 1.0* | 1.0 ± 0.2* | 1.3 ± 0.3* | 1.2 ± 0.5* |
| Body weight (g) | | | | |
| Control | 219 ± 6 | 235 ± 23 | 330 ± 19 | 345 ± 33 |
| Diabetic | 204 ± 9** | 225 ± 17 | 213 ± 49** | 267 ± 39** |
| Kidney weight (g) | | | | |
| Control | 0.84 ± 0.09 | 0.98 ± 0.08 | 1.12 ± 0.12 | 1.00 ± 0.09 |
| Diabetic | 0.88 ± 0.10 | 0.94 ± 0.09 | 1.11 ± 0.18 | 1.37 ± 0.21† |
| Kidney/100 g body weight | | | | |
| Control | 0.38 ± 0.04 | 0.41 ± 0.03 | 0.34 ± 0.03 | 0.29 ± 0.03 |
| Diabetic | 0.43 ± 0.05 | 0.42 ± 0.04 | 0.52 ± 0.08† | 0.51 ± 0.08† |

*Note.* six to eight animals were used in each group at the indicated times. ND, not determined.
*$P < .001$ compared with control animals; **$P < .01$ compared with control animals; †$P < .05$ compared with control animals.

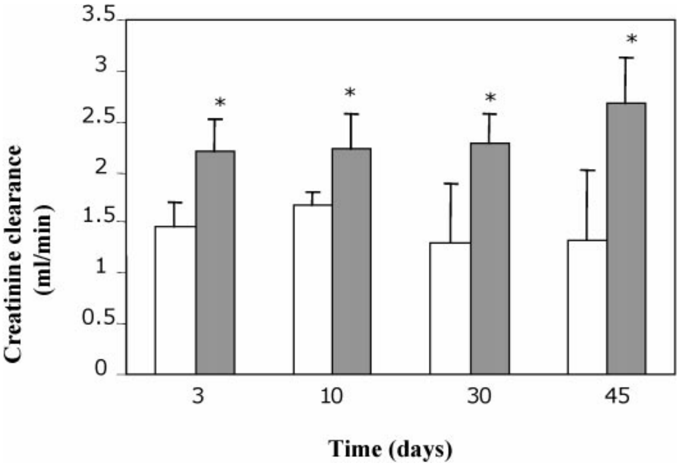

**FIGURE 1**
Creatinine clearance in control and mild STZ diabetic animals. At the indicated time, nondiabetic (□) and diabetic animals (■) were placed in metabolic cages for 48 hours of adaptation, during which 24-hour urine specimens were collected. Blood samples were also collected from tail vein at the end of urine collection. Results are expressed as mean + SE from 8 animals in each group. $^*P < .05$ compared with nondiabetic animals.

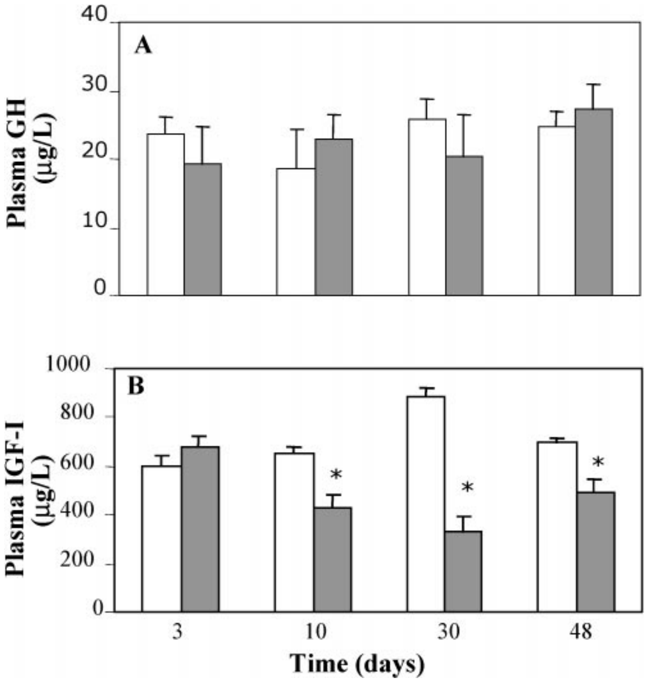

**FIGURE 2**
Plasma GH and IGF-I levels in control and mild STZ diabetic animals. Plasma GH (*A*) was measured by radioimmunoassay. Plasma IGF-I (*B*) was measured after extraction in ethanol–acetic acid as described in Materials and Methods. Results are expressed as mean + SE from 6 nondiabetic (□) and diabetic (■) animals in each group. $^*P < .01$ compared with control animals.

Despite the close to normal levels of insulin, the low STZ dose animals showed signs of early renal changes. Creatinine clearance (CrCl) was significantly elevated at day 3, reaching a 2-fold increase when measured at 45 days after the induction of diabetes, indicating that the alterations in kidney function were progressive (Figure 1). In contrast to the rapid elevation in CrCl, the increase in kidney size and in kidney/body weight ratio was delayed, with significant differences occurring only between 30 and 48 days after the induction of diabetes (Table 1).

The temporal discordance between changes in the CrCl and renal hypertrophy prompted us to study the expression of IGF-I and its binding proteins in both the systemic circulation and in the kidney in animals with mild hypoinsulinemia. Both protein and gene expression of the different components of the GH–IGF-I axis were measured.

Plasma GH levels in mildly diabetic animals remained similar to the values obtained in the control animals during the entire course of the study (Figure 2*A*). Plasma IGF-I levels were also unchanged after 3 days but were reduced by 34%, 63%, and 30% after 10, 30, and 48 days after the induction of diabetes, respectively (Figure 2*B*). Plasma 30-kDa IGFBPs (IGFBP-1/2) were unchanged during the entire course of the study (data not shown), while IGFBP-3 and IGFBP-4, similarly to serum IGF-I, were unchanged after 3 days but significantly declined at day 10 and remained reduced during the entire course of the study (Figure 3*A*, *B*).

In the kidney, IGFBP-1 mRNA levels were unchanged after 3 days but were elevated by 103% and 40%, 10 and 30 days after the induction of diabetes, respectively (Figure 4). The increase in kidney IGFBP-1 gene expression was associated with a delayed increase in kidney IGF-I, somewhat prolonged, and attenuated in the mild hypoinsulinemic rats. IGF-I was increased by 10% to 20%, 10 and 30 days after the induction of diabetes, respectively, compared with nondiabetic animals (Figure 5). Kidney 30-kDa (IGFBP-1/2), IGFBP-3, and IGFBP-4 were unchanged after 3, 10, and 30 days, but increased significantly at day 48 after the induction of diabetes (Figure 6*A*, *B*, *C*).

To evaluate whether the increase in renal growth in the mild hypoinsulinemic STZ diabetic rats was proportional to the insulin/glucose ratio and the kidney IGF system, a correlation study was carried out. A significant correlation between kidney/body weight ratio and insulin/glucose ratio ($r = .59$, $P < .05$) (Figure 7*A*) was found. This correlation tended to be stronger ($r = .68$, $P < .05$) in the animals after 30 and 48 days of diabetes, at the time when mild hypoinsulinemia was shown to increase renal growth (Figure 7*B*). To search which factor of the kidney IGF system can positively predict and explain the changes in kidney/body weight ratio, a correlation study was performed. Kidney IGF-I levels and IGFBP-1 mRNA correlated

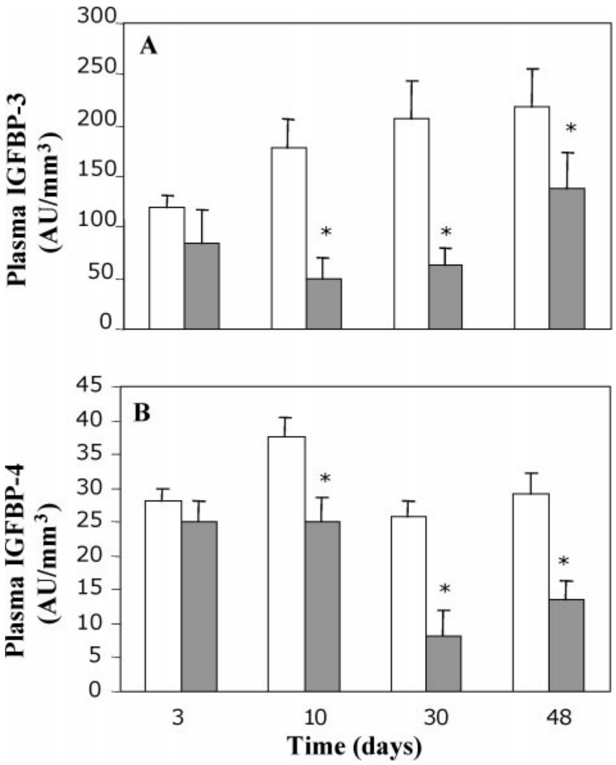

**FIGURE 3**
Plasma IGFBP-3 and IGFBP-4 levels in control and mild diabetic animals. Plasma IGFBP-3 (*A*) or IGFBP-4 (*B*) were determined in the indicated times using Western ligand blotting as described in Materials and Methods. Results are expressed as mean + SE from 6 nondiabetic (□) and diabetic (■) animals in each group. $^{*}P < .01$ compared with control animals.

significantly with renal hypertrophy ($r = .57$ and $r = .43$, respectively, $P < .05$) (Figure 8*A*, *B*). A mild correlation was also found between kidney growth and kidney 30-kDa IGFBPs (IGFBP-1/2) and IGFBP-4 ($P < .05$) (Figure 9*A*, *C*), without significant correlation with the kidney IGFBP-3 levels ($r = .27$) (Figure 9*B*).

### DISCUSSION

In our study, we found that mildly hypoinsulinemic animals developed renal hyperfiltration within 3 days of diabetes, whereas an increase in kidney size and the kidney/body weight ratio, markers for early diabetic kidney disease, were delayed, with significant differences occurring only between 30 and 48 days after the onset of diabetes. This is in contrast to severely hypoinsulinemic rats given higher doses of STZ, in which renomegaly developed already after 1 week of diabetes [18]. Plasma GH levels in diabetic animals were unchanged during our study. This is in distinction to studies of others [19, 20] showing a pronounced drop in GH levels in severely diabetic rats. Plasma IGF-I levels were diminished significantly on day

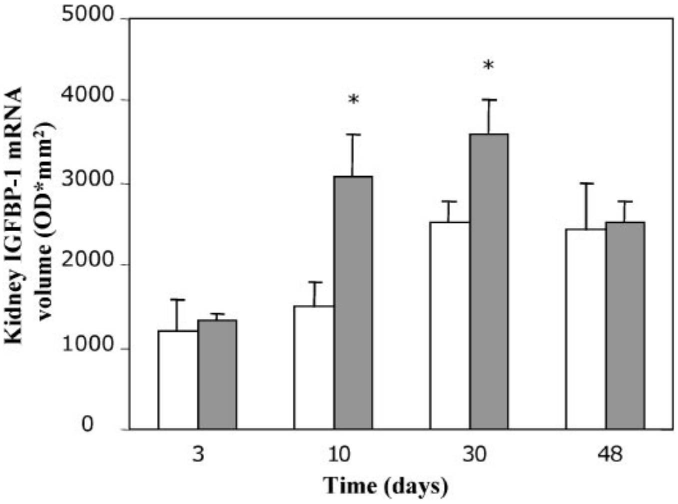

**FIGURE 4**
Kidney IGFBP-1 mRNA levels in nondiabetic and mild STZ diabetic animals. Total kidney RNA from the 2 groups was prepared, electrophoresed (20 $\mu$g), transferred, and hybridized as described in Materials and Methods. Relative mRNA amounts of IGFBP-1 was determined by densitometry and expressed as means + SE in bar graphs from 6 nondiabetic (□) and diabetic (■) animals in each group. $^{*}P < .03$ compared with control animals.

10, similar to the pattern seen in more severe diabetes, in which the decline in GH levels is paralleled by a fall in serum IGF-I beginning 5 to 7 days after the onset of diabetes [8, 18]. The fact that a decline in IGF-I occurs despite the lack of changes in GH levels suggests that there is a block in hepatic IGF-I production

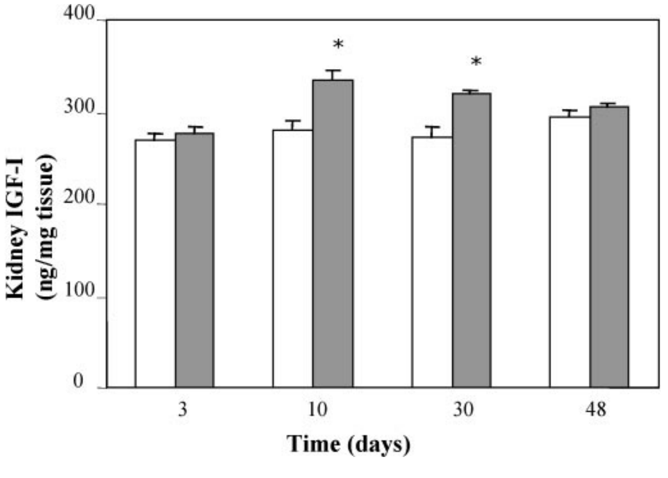

**FIGURE 5**
Kidney IGF-I levels in control and mild STZ diabetic animals. Kidney IGF-I extraction and measurement performed as described in Materials and Methods. Values mean + SE from 6 nondiabetic (□) and diabetic (■) animals in each group. $^{*}P < .05$ compared with control animals.

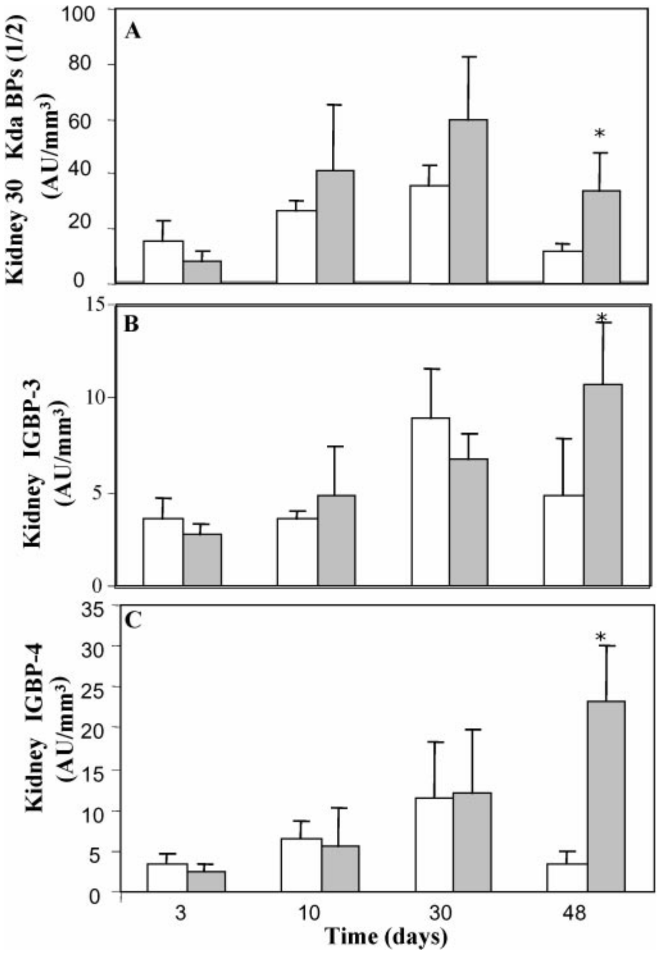

**FIGURE 6**

Kidney IGFBPs protein levels in control and mild STZ diabetic rats. Total kidney proteins (200 mg) from the 2 groups were subjected to SDS-PAGE on 10% polyacrylamide gels, transferred to nitrocellulose, and incubated with [$^{125}$I]-IGF-I as described in Materials and Methods. Specificity of the 30-kDa IGFBPs (IGFBP-1/2) (*A*), IGFBP-3 (*B*), and IGFBP-4 (*C*) bands confirmed by competitive coincubation with unlabeled IGF-II. Results of the densitometric analysis are expressed as mean + SE from 6 nondiabetic (□) and diabetic (■) animals in each group. $^{*}P < .02$ compared with control animals.

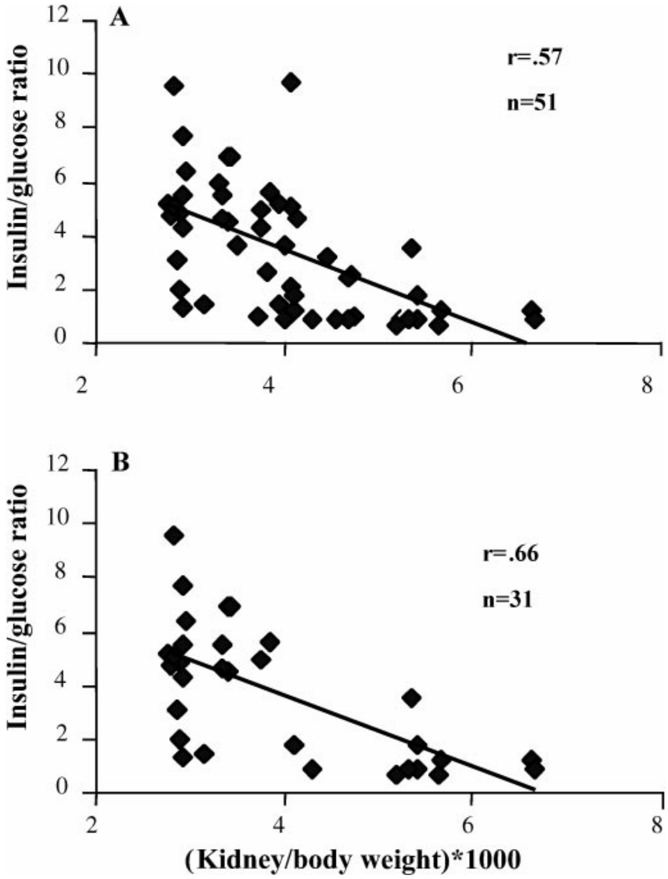

**FIGURE 7**

Correlation between insulin/glucose ratio and kidney/body weight ratio in all animals (*A*), or the 2 groups after 30 and 48 days of induction of diabetes (*B*).

or increased clearance of serum IGF-I, even in the presence of mild hypoinsulinemia. The decline in serum IGF-I levels have been attributed to the decreased transcription rate of the IGF-I gene in the liver [21]. This is consistent with the findings of other workers who found that providing GH to hypopituitary- or GH-deficient rats failed to increase IGF-I levels in diabetic rats [8, 22]. Plasma IGFBP-3 and IGFBP-4 declined significantly in the diabetic animals from day 10. This pattern is also similar to what can be found in severe diabetic rats [18]. IGFBP-3 is the major binding protein in the plasma and its decline parallels that of plasma IGF-I. The regulation of IGFBP-3 in diabetes has been studied extensively and found to be related to multiple factors, including insulin, IGF-I, GH, and glucocorticoids.

The discrepancy in time course between the rise in CrCl and renomegaly raises the question as to whether different mechanisms are involved in the development of kidney disease. In severe experimental diabetes, the rapid increase in kidney size and glomerular hypertrophy is associated with a transient increase in renal IGF-I concentrations that peak at 24 to 48 hours and subsequently return to normal by about 4 days [6]. Experimental evidence suggests that the increase in IGF-I is responsible for renal hypertrophy, as infusion of IGF-I accelerated diabetic hypertrophy [23]. Diabetic GH-deficient dwarf rats do not show a significant increase in renal IGF-I, with renal hypertrophy slower and milder [8]. IGFBP-1 increases significantly in the renal cortex after the induction of experimental diabetes, and it has been suggested that IGFBP-1 mediates the effects of IGF-I by enhancing delivery of IGF-I to kidney cell surface. Changes in the level of this binding protein may be significant in the development of diabetic kidney disease [2].

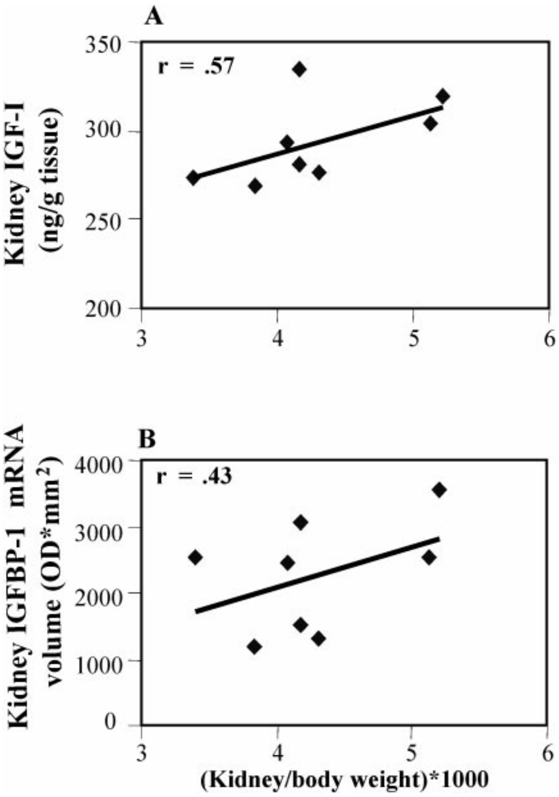

**FIGURE 8**
Correlation between the mean of kidney/body weight ratio and the mean of kidney IGF-I (*A*) and mean of IGFBP-1 mRNA (*B*) in the control and diabetic groups.

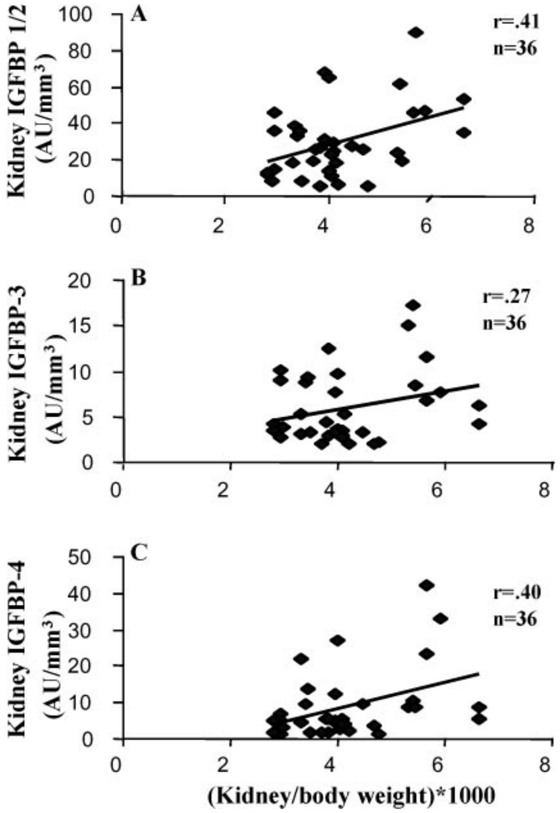

**FIGURE 9**
Correlation between kidney/body weight ratio and kidney IGFBP-1/2 (*A*), IGFBP-3 (*B*), and IGFBP-4 (*C*) in control and diabetic rats.

Comparing the data from mild hypoinsulinemic rats with diabetic dwarf rats provides an interesting contrast. Severely diabetic rats deficient in GH have an early-attenuated increase in IGF-I (which is blocked if insulin is administered) that returns to normal at day 4 [8]. In contrast, the increase in renal IGF-I in mildly hypoinsulinemic animals with an intact GH/IGF-I axis is delayed and prolonged. This indicates that the metabolic alterations associated with the diabetic state may regulate renal IGF-I levels both via the systemic GH/IGF-I axis and through direct effects on renal IGF-I levels, possibly by modulation of binding proteins. In the case of mildly hypoinsulinemic rats, it is probable that the delay in renomegaly is due to the fact that there is sufficient residual insulin secretion to blunt the significant and rapid increase in renal IGF-I that is seen in severe diabetes. The mechanism for the increase in renal IGF-I in mild hypoinsulinemia may be different from that in severe hypoinsulinemia. In mildly hypoinsulinemic animals, there is a significant increase in renal IGFBP-3 and IGFBP-4 seen at 48 days. This is in contrast to previous findings where no significant increase was seen in IGFBPs in long-term severe diabetes [24, 25]. Our data also suggest that there are other factors besides changes in renal IGF-I levels that impact on diabetic kidney disease. Changes in renal function occur early even in mild diabetes, despite that the IGF-I system remains unchanged. The elevated CrCl seen in these animals may result from either high glucose altering intraglomerular pressure or IGF-I–independent mechanisms such as the nitric oxide system [26]. Data from models of spontaneously developing diabetes studied in other laboratories also indicate that there may be different effects on either the morphology or function of the kidney, depending on whether the animal is severely diabetic and the type of diabetes (i.e., type 1 or type 2 diabetes) [25].

The traditional model of diabetic kidney disease employs a severely hypoinsulinemic animal in which changes in kidney morphology and kidney function occur very early, simultaneous to the alterations in the levels of systemic and renal IGF-I. It is probable that the diabetic kidney disease in clinical diabetes is more similar to that of mild hypoinsulinemia and that the mechanisms for type 2 diabetes may be entirely different because of the elevated levels of insulin. To develop a better understanding of diabetic kidney disease, it will be necessary to perform comprehensive studies on a variety of diabetic models to determine the exact mechanisms by which alterations in the metabolic environment transduce changes leading to the development of diabetic kidney disease.

### REFERENCES

[1] Raz, I., and Wexler, I. D. (1999) Extrarenal complications of the type 2 diabetes patient with diabetic nephropathy. In: *Nephropathy in Type 2 Diabetes,* Edited by Ritz, E., and Rychlik, I., pp. 158–194. Oxford, UK, Oxford University Press.

[2] Flyvbjerg, A. (1997) Role of growth hormone, insulin-like growth factors (IGFs) and IGF-binding proteins in the renal complications of diabetes. *Kidney Int. Suppl.,* **60,** S12–S19.

[3] Flyvbjerg, A., Hill, C., and Logan, A. (1999) Pathophysiological role of growth factors in diabetic kidney disease: Focus on innovative therapy. *Trends Endocrin. Metab.,* **10,** 267–272.

[4] Abboud, H. E. (1997) Growth factors and diabetic nephrology: An overview. *Kidney Int. Suppl.,* **60,** S3–S6.

[5] Winter, R. J., Phillips, L. S., Klein, M. N., Traisman, H. S., and Green, O. C. (1979) Somatomedin activity and diabetic control in children with insulin-dependent diabetes. *Diabetes,* **28,** 952–954.

[6] Flyvbjerg, A., Bornfeldt, K. E., Marshall, S. M., Arnqvist, H. J., and Orskov, H. (1990) Kidney IGF-I mRNA in initial renal hypertrophy in experimental diabetes in rats. *Diabetologia,* **33,** 334–338.

[7] Unterman, T. G., Jentel, J. J., Oehler, D. T., Lacson, R. G., and Hofert, J. F. (1993) Effects of glucocorticoids on circulating levels and hepatic expression of insulin-like growth factor (IGF)-binding proteins and IGF-I in the adrenalectomized streptozotocin-diabetic rat. *Endocrinology,* **133,** 2531–2539.

[8] Flyvbjerg, A., Frystyk, J., Osterby, R., and Orskov, H. (1992) Kidney IGF-I and renal hypertrophy in GH-deficient diabetic dwarf rats. *Am. J. Physiol.,* **262,** E956–E962.

[9] Flyvbjerg, A., and Orskov H. (1990) Kidney tissue insulin-like growth factor I and initial renal growth in diabetic rats: Relation to severity of diabetes. *Acta Endocrinol. Copenh.,* **122,** 374–378.

[10] D'Ercole, A. J., Stiles, A. D., and Underwood, L. E. (1984) Tissue concentrations of somatomedin C: Further evidence for multiple sites of synthesis and paracrine or autocrine mechanisms of action. *Proc. Natl. Acad. Sci. U.S.A.,* **81,** 935–939.

[11] Weiss, O., Anner, H., Nephesh, I., Alayoff, A., Bursztyn, M., and Raz, I. (1995) Insulin-like growth factor-I (IGF-I) and IGF-I receptor gene expression in the kidney of the chronically hypoinsulinemic rat and hyperinsulinemic rat. *Metabolism,* **44,** 982–986.

[12] Pugliese, G., Pricci, F., Locuratolo, N., Romeo, G., Romano, G., Giannini, S., Cresci, B., Galli, G., Rotella, C. M., and Di Mario, U. (1996) Increased activity of the insulin-like growth factor system in mesangial cells cultured in high glucose conditions. Relation to glucose-enhanced extracellular matrix production. *Diabetologia,* **39,** 775–784.

[13] Narayanan, S., and Appleton, H. D. (1980) Creatinine: A review. *Clin. Chem.,* **26,** 1119–1126.

[14] Furlanetto, R. W., and Marino, J. M. (1987) Radioimmunoassay of somatomedin C/insulin-like growth factor I. *Methods Enzymol.,* **146,** 216–226.

[15] Flyvbjerg, A., Kessler, U., Dorka, B., Funk, B., Orskov, H., and Kiess, W. (1992) Transient increase in renal insulin-like growth factor binding proteins during initial kidney hypertrophy in experimental diabetes in rats. *Diabetologia,* **35,** 589–593.

[16] Symon, Z., Fuchs, S., Agmon, Y., Weiss, O., Nephesh, I., Moshe, R., Brezis, M., Flyvbjerg, A., and Raz, I. (1998) The endogenous insulin-like growth factor system in radiocontrast nephropathy. *Am. J. Physiol.* **274,** F490–F497.

[17] Werner, H., Woloschak, M., Adamo, M., Shen-Orr, Z., Roberts, C. T. J., and LeRoith, D. (1989) Developmental regulation of the rat insulin-like growth factor I receptor gene. *Proc. Natl. Acad. Sci. U.S.A.,* **86,** 7451–7455.

[18] Raz, I., Rubinger, D., Popovtzer, M., Gronbaek, H., Weiss, O., and Flyvbjerg, A. (1998) Octreotide prevents the early increase in renal insulin-like growth factor binding protein 1 in streptozotocin diabetic rats. *Diabetes,* **47,** 924–930.

[19] Tannenbaum, G. (1981) Growth hormone secretion dynamics in streptozotocin diabetes: Evidence of a role for endogenous somatostatin. *Endocrinology,* **108,** 76–82.

[20] Robinson, I. C., Clark, R. G., and Carlsson, L. M. (1987) Insulin, IGF-I and growth in diabetic rats. *Nature,* **326,** 549.

[21] Phillips, L. S., Pao, C. I., and Villafuerte, B. C. (1998) Molecular regulation of insulin-like growth factor-I and its principal binding protein, IGFBP-3. *Prog. Nucleic Acid Res. Mol. Biol.,* **60,** 195–265.

[22] Maes, M., Underwood, L. E., and Ketelslegers, J. M. (1986) Low serum somatomedin-C in insulin-dependent diabetes: Evidence for a postreceptor mechanism. *Endocrinology,* **118,** 377–382.

[23] Flyvbjerg, A., Bornfeldt, K. E., Orskov, H., and Arnqvist, H. J. (1991) Effect of insulin-like growth factor I infusion on renal hypertrophy in experimental diabetes mellitus in rats. *Diabetologia,* **34,** 715–720.

[24] Landau, D., Chin, E., Bondy, C., Domene, H., Roberts, C. T. J., Gronbaek, H., Flyvbjerg, A., and LeRoith, D. (1995) Expression of insulin-like growth factor binding proteins in the rat kidney: Effects of long-term diabetes. *Endocrinology,* **136,** 1835–1842.

[25] Flyvbjerg, A. (2000) Putative pathophysiological role of growth factors and cytokines in experimental diabetic kidney disease. *Diabetologia,* **43,** 1205–1223.

[26] Keynan, S., Hirshberg, B., Levin-Iaina, N., Wexler, I. D., Dahan, R., Reinhartz, E., Ovadia, H., Wollman, Y., Chernihovskey, T., Iaina, A., and Raz, I. (2000) Renal nitric oxide production during the early phase of experimental diabetes mellitus. *Kidney Int.,* **58,** 740–747.

